# Hybrid 0D Antimony Halides as Air‐Stable Luminophores for High‐Spatial‐Resolution Remote Thermography

**DOI:** 10.1002/adma.202007355

**Published:** 2021-01-22

**Authors:** Viktoriia Morad, Sergii Yakunin, Bogdan M. Benin, Yevhen Shynkarenko, Matthias J. Grotevent, Ivan Shorubalko, Simon C. Boehme, Maksym V. Kovalenko

**Affiliations:** ^1^ Laboratory of Inorganic Chemistry Department of Chemistry and Applied Bioscience ETH Zürich Vladimir Prelog Weg 1 Zürich CH‐8093 Switzerland; ^2^ Laboratory for Thin Films and Photovoltaics Empa – Swiss Federal Laboratories for Materials Science and Technology Überlandstrasse 129 Dübendorf CH‐8600 Switzerland; ^3^ Laboratory for Transport at Nanoscale Interfaces Empa – Swiss Federal Laboratories for Materials Science and Technology Überlandstrasse 129 Dübendorf CH‐8600 Switzerland

**Keywords:** metal halide luminescence, remote thermography, thermographic phosphors

## Abstract

Luminescent organic–inorganic low‐dimensional ns^2^ metal halides are of rising interest as thermographic phosphors. The intrinsic nature of the excitonic self‐trapping provides for reliable temperature sensing due to the existence of a temperature range, typically 50–100 K wide, in which the luminescence lifetimes (and quantum yields) are steeply temperature‐dependent. This sensitivity range can be adjusted from cryogenic temperatures to above room temperature by structural engineering, thus enabling diverse thermometric and thermographic applications ranging from protein crystallography to diagnostics in microelectronics. Owing to the stable oxidation state of Sb^3+^, Sb(III)‐based halides are far more attractive than all major non‐heavy‐metal alternatives (Sn‐, Ge‐, Bi‐based halides). In this work, the relationship between the luminescence characteristics and crystal structure and microstructure of TPP_2_SbBr_5_ (TPP **=** tetraphenylphosphonium) is established, and then its potential is showcased as environmentally stable and robust phosphor for remote thermography. The material is easily processable into thin films, which is highly beneficial for high‐spatial‐resolution remote thermography. In particular, a compelling combination of high spatial resolution (1 **µ**m) and high thermometric precision (high specific sensitivities of 0.03–0.04 K^−1^) is demonstrated by fluorescence‐lifetime imaging of a heated resistive pattern on a flat substrate, covered with a solution‐spun film of TPP_2_SbBr_5_.

## Graphical abstract

Luminescent organic–inorganic low‐dimensional ns^2^ metal halides are increasingly explored as thermal phosphors for high‐resolution remote thermography and thermometry due to the high thermal sensitivity of their radiative lifetime. Thin films of such hybrids, conformally coating the surface of interest, can map the temperature with a spatial precision as high as 1 μm using fluorescence‐lifetime imaging.

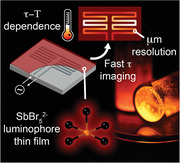

The rapid technological advances of the last century have resulted in new challenges in the area of temperature sensing. Accurate, remote, contactless, and real‐time micro‐ and nanoscale temperature mapping is of great demand in cell imaging, micro‐ and nanofluidics, and integrated circuit design,^[^ ^1^, ^2^, ^3^, ^4^, ^5^, ^6^, ^7^, ^8^, ^9^, ^10^, ^11^ ^]^ in which these stringent requirements necessitate the use of optical methods. These are typically separated into three primary categories: infrared (IR) bolometry, IR direct detection, and remote optical/fluorescence thermography. Of these, IR bolometric methods, such as those found in commercial devices, are the most common due to their excellent thermal resolution (10^−1^ K). However, the long IR wavelengths of the black‐body radiation that is to be detected result in inherently low spatial resolutions of ≈10 µm for room‐temperature (RT) objects, which is expected by the Abbe diffraction limit. Detection of IR light also suffers from a lack of compatibility with widespread optical components as a result of absorption.^[^ ^12^, ^13^ ^]^ Alternatively, remote optical methods operating in the visible region, for instance, by measuring fluorescence intensity or decay times,^[^ ^14^ ^]^ have attained high thermal resolution and may potentially offer higher spatial resolution due to a lower diffraction limit, and transparency in common media such as water and glass.^[^ ^13^, ^15^, ^16^ ^]^ Intensity‐based quantifications are, however, prone to errors due to light scattering (sample topology, phosphor particle morphology, etc.), inhomogeneous phosphor distribution, inhomogeneous phosphor speciation or batch‐to‐batch variability, etc. While fluorescence‐lifetime‐based thermography is inherently immune to many of these limitations, its deployment is often hampered by the availability of a phosphor that suits the specific requirements for a particular application. Our herein presented study concerns the exploration of novel thermoluminophores for high spatial and thermal resolution thermography at temperatures around RT. In this context, we find that known thermoluminophores, namely, organic dyes, polymers, quantum dots, rare‐earth‐doped metal‐oxides,^[^ ^17^, ^18^, ^19^, ^20^, ^21^, ^22^, ^23^, ^24^, ^25^ ^]^ face limitations such as complexity in materials fabrication or thin‐film deposition, durability and robustness, or unsuited optical characteristics for a specific temperature range or common detection methods.

Recent years have seen revived interest in luminescent metal halides, in particular, 0D halides of ns^2^‐ions (e.g., Ge^2+^, Sn^2+^, Pb^2+^, Sb^3+^, Bi^3+^, Te^4+^, Cu^+^).^[^ ^26^, ^27^, ^28^, ^29^, ^30^, ^31^, ^32^, ^33^, ^34^, ^35^, ^36^, ^37^, ^38^, ^39^, ^40^, ^41^, ^42^, ^43^, ^44^ ^]^ These typically exhibit color‐tunable, broadband emission from self‐trapped excitons (STE)^[^ ^45^, ^46^, ^47^ ^]^ often with high photoluminescence quantum yield (PLQY) at RT. While these halides are typically discussed in the context of utility‐scale applications such as solid‐state lighting,^[^ ^48^ ^]^ we have recently demonstrated their use as thermoluminophores in remote thermometry and millimeter‐scale thermography owing to the steep temperature dependence of their luminescence decay rates.^[^ ^49^ ^]^ The temperature distribution of a surface can therefore be mapped by conducting fluorescence‐lifetime imaging (FLIM, **Figure** **1**a). The emission lifetime–temperature (τ–*T*) dependence (Figure 1b), to be reliably applied in thermography, has to be intrinsic to the material of a given structure and composition and without batch‐to‐batch variability. The steep τ–*T* dependence in STE emitters arises from thermal activation of phonon‐assisted nonradiative channels for the detrapping of STEs that become competitive with radiative STE recombination within a specific temperature range. This temperature range, in which both PLQY and τ decrease until the emission vanishes, is referred to as the sensitivity range of the thermoluminophore, and it typically spans a range of 100 K, which can be engineered for a specific application by adjusting the crystal structure and composition. Regardless of the physics of STE quenching, τ–*T* dependence can be fitted with a sigmoid law (Figure 1b) that shows that the optimal operating region is around *T*_q_ (temperature at which emission is quenched to 50% of its maximum value). Practically speaking, compounds with moderate RT‐PLQYs (*T*_q_ close to RT) may be considered for a broad range of thermal sensing applications, such as bio‐imaging (physiological temperatures, i.e., 20–50 °C), heat distribution in electronics (RT‐120 °C), etc.

**Figure 1:**
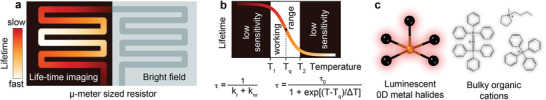
a) A schematic depiction of a flat substrate with micrometer‐sized resistive pattern. When the current flows through the pattern, the temperature can be probed in the lifetime imaging experiment with high thermal and spatial resolution. b) A Boltzmann sigmoid function used to describe typical temperature dependence of the emission decay lifetime τ of the thermal phosphors. The working range of the phosphor is defined as the linear region around the 50% quenched point *T*_q_. c) Molecular geometry of the structurally isolated SbX_5_^2−^ center and typical organic cations that can be used to form organic–inorganic hybrid compounds.

An important characteristic of STE‐emissive ns^2^‐metal halide luminophores is their moderate‐to‐slow radiative rates in a nonquenched state, which are then drastically increased by up to two orders of magnitude with increasing temperature, thus yielding high specific detection sensitivities (up to 0.06 °C^−1^).^[^ ^49^ ^]^ The respective τ‐values are typically in the microsecond‐to‐nanosecond range, making for a good match to various inexpensive PL lifetime measurement schemes.^[^ ^50^, ^51^ ^]^ While we have observed these characteristics and attained tunable sensitivity ranges with several hybrid organic–inorganic Sn(II) halides and the fully inorganic Cs_4_SnBr_6_,^[^ ^49^ ^]^ these compounds inherently suffer from oxidation in air, making their use in high‐spatial‐resolution thermography unfeasible. In particular, the retention and successful detection of the thermal gradients present within the sample requires a homogeneous and conformal coating with minute amounts of luminophore. The use of encapsulating coating layers atop the luminophores is then required, which is orthogonal to the purpose of faithful retention of the thermal gradients and can be prohibitively laborious. Therefore, air‐durable alternatives, such as the Sb(III)‐halides,^[^ ^47^, ^52^ ^]^ are required. Such materials usually comprise isolated SbX_5_^2−^ anions separated by bulky organic cations (Figure 1c). The vast library of 0D Sb(III)‐halides allows the *T*_q_ to be adjusted from below 200 K (iodides), to around RT (bromides), and above 400 K (chlorides).^[^ ^45^, ^52^, ^53^, ^54^, ^55^, ^56^, ^57^, ^58^, ^59^, ^60^, ^61^, ^62^, ^63^, ^64^, ^65^, ^66^, ^67^, ^68^, ^69^, ^70^ ^]^

Targeting a sensitivity range around RT, we focus on tetraphenylphosphonium (TPP) Sb(III) pentabromide (TPP_2_SbBr_5_). Two synthetic routes were pursued: i) by dissolving stoichiometric amounts of TPPBr and SbBr_3_ salts in a polar organic solvent (dichloromethane, DCM) and using diethyl ether (Et_2_O) as an antisolvent, or ii) melting the TPPBr and SbBr_3_ in a stoichiometric ratio at 230 °C (**Figure** **2**a; see the Experimental Section for details). TPP_2_SbBr_5_ is obtained in either a disordered (route i) or ordered (route ii) phase (Figure 2b and Figure S1, Supporting Information), both without organic solvent or water molecules in their crystal structures. The disordered phase is characterized by the static disorder of the alignment of SbBr_5_^2−^ square pyramids, resulting in 50% occupancy of axial positions by Br atoms, whereas in the ordered phase, the static disorder is eliminated, and Br atoms occupy 100% of axial position. The ordering of the disordered phase can be observed at 186 °C and is an irreversible process (Figure S2, Supporting Information). Such an interplay between kinetic and thermodynamic processes has been previously observed in Bi(III)‐based organic–inorganic halides,^[^ ^71^ ^]^ and, to the best of our knowledge, this is the first example for Sb(III) halides. The softness of ns^2^‐metal halides culminates in their hybrid organic–inorganic variants, as seen not only from low melting points (<300 °C) but also from the increasingly evident impact of the structural disorder on the optical characteristics; for Sb(III) halides, see examples in refs. ^[^ ^69^, ^72^, ^73^ ^]^. In the following, we establish and rationalize such effects for TPP_2_SbBr_5_ and showcase their importance for applications in high‐spatial‐resolution thermography.

**Figure 2:**
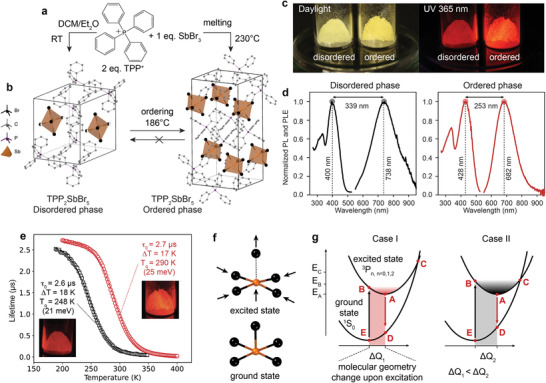
a) A scheme depicting chemical and phase transformations occurring in the TPPBr‐SbBr_3_ system. A wet synthetic route from the solvent–antisolvent method (DCM‐Et_2_O) results in the TPP_2_SbBr_5_ product which is a disordered phase (half‐filled Br atoms indicate 50% occupancy). The ordered phase can be obtained either by melting TPPBr and SbBr_3_ in stoichiometric ratio above 230 °C or by heating the disordered phase above 186 °C. After heat treatment, the cooled ordered phase does not transform back to the disordered phase, indicating that the ordered phase is thermodynamically stable at room temperature. b) Crystallographic unit cells of the ordered and disordered phases of TPP_2_SbBr_5_. c) Photos of the corresponding disordered and ordered TPP_2_SbBr_5_ powders under daylight and UV excitation (365 nm). d) PLE and PL spectra of the ordered and disordered phases, demonstrating characteristic broad and largely Stokes shifted emission from isolated center. e) Lifetime–temperature dependence for disordered (black squares) and ordered (red circles) TPP_2_SbBr_5_ powders and corresponding Boltzmann–Sigmoid fits with fitting parameters. f) Depiction of the local geometry distortion occurring upon excitation in SbBr_5_^2−^. The axial Sb—Br bond is elongated, and equatorial Sb—Br bonds are shortened; the Sb atom experiences out‐of‐plane distortion. g) Seitz–Mott single configurational coordinate model depicting processes involved in radiative and nonradiative exciton recombination pathways in organic–inorganic Sb(III) halide hybrids. The radiative process involves excitation (vertical transition E–B) with subsequent relaxation to the excited state geometry (horizontal transition B–A involving phonons) and emission (vertical transition A–D) with relaxation to the ground state geometry (horizontal transition D–E). A nonradiative process occurs when the system can reach the crossing point of two parabolas (horizontal transition B–C) and relax back to ground state E without emission of a photon (horizontal transition C–E). Depending on the excited state parabola offset from the ground state (Δ*Q*, shaded region), the crossing point C can occur energetically close to point B or much above. The latter case results in higher‐temperature quenching point *T*_q_ as the phonon modes involved in transition B–C need to be activated.

Both ordered and disordered TPP_2_SbBr_5_ are characterized by PL excitation (PLE) extending from below 250 nm to about 500 nm and largely Stokes‐shifted red emission (Figure 2c,d and Figure S3, Supporting Information). On the contrary, Sn(II)‐bromide luminophores can be excited only at wavelengths below 400 nm,^[^ ^29^, ^74^ ^]^ posing a challenge for both the excitation‐detection instrumentation and some applications (increased autofluorescence background in bio‐imaging). The disordered TPP_2_SbBr_5_ phase is characterized by PLE shifted to higher energies, whereas the PL band is shifted to lower energies as compared to the ordered phase (Figure 2d). This results in a significant increase of the Stokes shift from 1.08 eV (253 nm) in the ordered phase to 1.42 eV (339 nm) in the disordered phase. At RT, the disordered phase exhibits lower PLQY (3.3%) and *T*_q_ (248 K) than the ordered phase (PLQY = 33%; see Table S3, Supporting Information; *T*_q_ = 290 K; see τ–*T* dependence in Figure 2e). These properties together with the Stokes shift are intricately related to the ground and excited state molecular geometry. For 0D emissive centers, the electronic states are highly localized.^[^ ^29^, ^39^, ^45^ ^]^ Excitation leads to a large structural distortion, as depicted in Figure 2f. An atomistic picture of the involved processes can be depicted using a single‐coordinate configurational diagram model, where ground and excited states are represented by parabolic potential functions (harmonic for the simplicity, Figure 2g).^[^ ^75^ ^]^ The abscissa depicts the change in the bond length or atomic coordinates; energy is placed on the ordinate. The minimum of the parabola represents the equilibrium bond distance, which is different for the ground and excited states. A Seitz–Mott model is adopted to rationalize the optical properties.^[^ ^76^ ^]^ Thermal activation is required to enable the nonradiative pathway and hence *T*_q_ scales with the energy difference between points C and B (Figure 2g). When this difference is small (case II), the emission will be nearly quenched already at RT. Likewise, the Stokes shift (Figure 2g, BE‐AD) increases with the molecular geometry change upon excitation (Figure 2g, shaded area related to Δ*Q*) and is thus larger for case II. We now connect the Seitz–Mott model to our experimental data. First, regarding the ordinate in Figure 2g, our optical spectroscopy data suggest that the ordered phase of TPP_2_SbBr_5_ classifies as case I, while the disordered phase as case II: both thermal quenching and Stokes shift are more pronounced in the latter. Second, regarding the abscissa in Figure 2g, we then use X‐ray‐based structure determination and density functional theory (DFT) calculations to confirm that the disordered phase (case II) indeed also exhibits a larger photoinduced structural distortion than the ordered phase (case I). The computed SbBr_5_^2−^ excited‐state geometries are comparable in both phases (with elongated axial Sb—Br bonds of about 3 Å and pronounced out‐of‐plane distortion of Sb; see the Experimental Section for details on the DFT calculations). The geometries of the ground state are, however, markedly different: the X‐ray‐based structure determination finds axial Sb—Br bond lengths of 2.38 and 2.53 Å in the disordered and ordered phase, respectively, and minor changes in the equatorial Sb—Br bonds (Tables S4–S7, Supporting Information). Thus, we conclude that photoexcitation causes a larger structural distortion in the disordered phase, which, in line with the Mott–Seitz model, results in the larger Stokes shift and lower temperature for thermal PL quenching. We note that the Seitz–Mott diagram also helps rationalizing other temperature‐dependent properties in 0D metal halides, such as the homogeneous broadening of PL and PLE bands (Figure S4, Supporting Information).

An important difference exists between Sn‐based thermal luminophores studied in the ref. ^[^ ^49^ ^]^ and the herein presented Sb‐based compounds, due to the more complex excited state structure in the latter, which arises from the nondegeneracy of the lowest unoccupied molecular orbital (LUMO) states in square pyramidal molecular geometry and exciton–phonon interactions.^[^ ^47^, ^68^, ^77^ ^]^ TPP_2_SbBr_5_ exhibits spectral variation in emission relaxation times (shorter relaxation times for shorter wavelengths and longer relaxation times for longer wavelengths, Figure S5a,c, Supporting Information) and non‐monoexponential behavior in the traces detected in narrow spectral windows (Figure S5b, Supporting Information), as previously demonstrated for other pnictogen halides.^[^ ^78^ ^]^

To test the TPP_2_SbBr_5_ as a thermal phosphor for high‐spatial‐resolution thermography, thin films were prepared by spin‐coating from the precursor solution containing TPPBr and SbBr_3_ in an organic solvent (CH_3_CN or DCM). The best homogeneity and uniformity can be achieved in the 150 nm thin film (Figure S6a,b, Supporting Information). Although very thin, such a film has sufficient absorbance (Figure S6c, Supporting Information). The film can be coated on a wide range of substrates including, but not limited to, glass, quartz, and silicon (see the Experimental Section for details). The as‐prepared film is close to the disordered phase from its optical properties, and the ordered phase can be achieved by annealing the film at higher temperatures or by slow aging over more than 24 h. However, after thermal annealing or aging, the ordered film exhibits far more grain boundaries, which is incompatible with the high spatial resolution that we aim to demonstrate (Figure S6d, Supporting Information). This is summarized in **Figure** **3**a. Eventually, for the FLIM experiment we have used a freshly deposited homogenous film on a flat glass substrate patterned with a small, micrometer‐scaled, indium tin oxide (ITO) pattern that mimics a structure similar to a microelectronic component. This pattern was made by photolithography with the smallest feature having a size of about 3 µm. The quality of the film before the FLIM experiment can be concluded from the homogenous PL detected from the film over the region of interest (Figure 3b). The τ–*T* dependence of such a film is plotted in Figure 3c. Although *T*_q_ is similar to the disordered bulk phase, τ_0_ is more than two times smaller. This could be an evidence that the film, due to its thickness, is even more disordered and hence more quenched. The differences in exciton dynamics between bulk and thin film morphology of low‐dimensional metal halides are relatively unknown, with the exception of a recent study by Thomaz et al. that points out the difference in excitation decay time in bulk and thin film forms of a corrugated lead(II) bromide compound.^[^ ^79^ ^]^ To further confirm the identity of the ordered and disordered films and to probe the local disorder, we have analyzed the lower frequencies of the Raman spectra (at RT) for the fresh and annealed films and compared them to those recorded for ordered and disordered bulk powders (Figure 3d). To analyze these spectra, we have simulated the phonon density of states plot at 300 K and identified the region where Sb and Br contribute the most, around 120–160 cm^−1^ (Figure S7, Supporting Information). The experimental spectra of the ordered bulk and ordered film are quite similar and feature sharp peaks. On the contrary, fresh thin film spectra match best with the disordered bulk phase, which along with the broadened Raman peaks and τ–*T* profile confirms the disordered state of the fresh film. As to the thermal performance, specific sensitivities of the ordered film and disordered bulk are rather comparable, spanning from 0.03 to 0.045 °C^−1^ in the ranges around RT (Figure S8, Supporting Information), which is at the higher end among those reported.^[^ ^80^ ^]^

**Figure 3:**
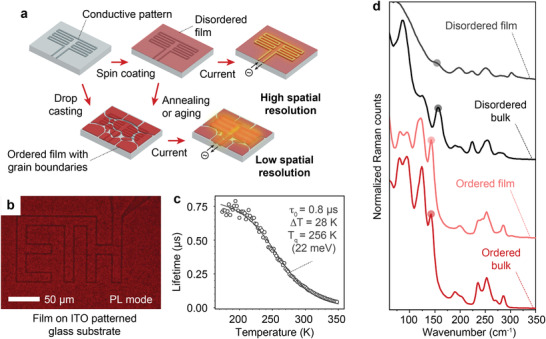
a) A schematic description of the TPP_2_SbBr_5_ phosphor disordered thin film (spin‐coated from polar organic solvent) and its subsequent transformation. The morphology of the film influences the outcome of the FLIM experiment: ordering of the thin film from annealing or aging is accompanied by formation of grain boundaries which lower the spatial resolution. b) Optical microscopy image (in PL detection mode) of a homogenous 150 nm thin film of TPP_2_SbBr_5_ (disordered phase) on a glass substrate and ITO resistive pattern. c) Lifetime–temperature dependence for the disordered thin film of TPP_2_SbBr_5_. d) Raman spectra for ordered and disordered bulk and thin film samples. Highlighted are the peaks which mostly originate from Sb and Br contribution in calculated phonon density of states.

**Figure** **4** demonstrates the ability of remote‐optical thermography to achieve high spatial resolution. With confocal microscopy equipped with FLIM and having diffraction‐limited resolution, the obtained thermographic resolution is determined by factors such as patterned feature size, heat distribution in the film, and the luminophore film thickness. A FLIM microscope acquires point‐by‐point lifetimes of the luminophore film that, with high sensitivity, reproduces the sample surface temperature. Without a current passing through the ITO pattern, the sample temperature is homogenous, and a careful measurement allows for an estimate of the lifetime value distribution. The FWHM of this distribution (Figure S9, Supporting Information) shows the pixel‐to‐pixel lifetime τ measurement variation for the instrument and for our recording conditions (including spatial resolution and exposition time). The resulting Δτ is about 2 ns, which is equivalent to about 1 K in Δ*T* as thermographic accuracy of our luminophores. When a current passes through the pattern, it generates local heat (recorded in low spatial resolution by microbolometer camera on Figure 4a). Figure 4b–e compares the performance of both disordered and ordered (obtained by thermal annealing) films in the FLIM experiment. The ordered film displays low spatial resolution due to large grains, which can also be seen in fluorescence images (Figure S10, Supporting Information). The temperature‐induced changes in the disordered film are much sharper (Figure 4e), as is also emphasized in Figure S11a (Supporting Information), where the corresponding lifetime measurement distributions (red curve from Figure S11b, Supporting Information) demonstrate the decrease of the emission relaxation times (more on the heating pattern, less in the surroundings of the patterned sample area). This is also demonstrated by the reversibility that is shown from the lifetime distributions (blue and green curves from Figure S11b, Supporting Information) recorded from a cold sample before and after heating. The spatial resolution of the imaged features from Figure 4e, estimated with a Gaussian fit (Figure 4c), is about 1 µm, far better than what can be achieved with remote detection of blackbody radiation at RT.

**Figure 4:**
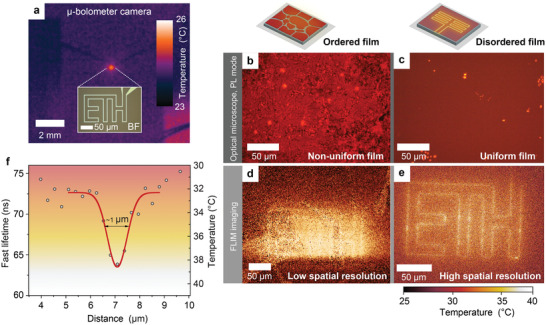
a) Image of the TPP_2_SbBr_5_ disordered thin film on the glass substrate with resistive pattern with a conventional micrometer bolometer camera. The inset image shows the bright‐field light microscopy image of the small features to demonstrate the scale. b,c) Optical (PL mode) of the disordered and ordered films of TPP_2_SbBr_5_ on the nonpatterned glass substrates, comparing the uniformity of the film. d,e) Confocal FLIM microscopy images of the disordered and ordered films of TPP_2_SbBr_5_ on the patterned ITO substrate with current running through the resistive pattern. The ordered (disordered) film results in low (high) spatial resolution. f) The pixel intensity for a line scan across one of the heated features in (e); a spatial resolution as high as about 1 µm can be estimated from a Gaussian fit.

The non‐monoexponential decay characteristics of TPP_2_SbBr_5_ add a degree of complexity to the analysis of PL lifetimes in FLIM experiments, which can drastically increase the time required to process thermal images. This issue can be avoided through the use of a simple 1/*e* evaluation (Figure S12 and Note S1, Supporting Information).

In conclusion, we demonstrate the untapped potential of Sb(III) 0D hybrid ns^2^‐metal halide organic–inorganic emitters as thermal phosphors for high‐spatial‐resolution thermography around RT ranges. Thin‐film Sb(III)‐based phosphors do not suffer from instability in air (Figure S13, Supporting Information), as opposed to Sn(II)‐based ones, for which prevention of oxidation is paramount at all steps (preparation, deposition, and use). Thin films of such halides harness their high specific thermal sensitivity (0.03–0.045 °C^−1^ for TPP_2_SbBr_5_) and allow for micrometer resolution of thermography. On the example of TPP_2_SbBr_5_, this work emphasizes the existence and necessitates the study of the differences in the structure and hence in the respective optical characteristics that occur when transitioning from the bulk to thin‐film forms of these soft halides. An unchartered research avenue is to attain higher spatial resolution using colloidal nanoparticles of STE‐emissive materials. Eventually, a vast library of STE‐emissive metal halide phosphors, accessible through facile solution synthesis and processing, will provide an a la carte selection of a suite of optical characteristics (PL and PLE bands, temperature sensitivity range, etc.) required for a specific thermometric and thermographic application and/or detection scheme.

## Experimental Section

*Synthesis of Bulk Powder of TPP_2_SbBr_5_*: In a typical synthetic procedure, TPPBr (0.2 mmol) and SbBr_3_ (0.1 mmol) were mixed in 2 mL of DCM at room temperature. The solution was filtered through 250 µm PTFE filter and injected into excess of Et_2_O (15 mL). The precipitate was further washed once with acetone and dried under reduced pressure overnight.

*Synthesis of Single Crystals (SC) of TPP_2_SbBr_5_*: SbBr_3_ (36.5 mg) and TPPBr (126.09 mg) dissolved in 3 mL of DCM and layered carefully with 3 mL of Et_2_O. After 1–2 d, light‐yellow crystals (0.5–1 mm) of the disordered phase were obtained. To obtain the single crystals of the ordered phase which were suitable for the SC X‐ray diffraction (XRD) analysis, crystals of the disordered phase were very slowly heated (at a rate about 1 °C min^−1^) to 185 °C in a glass vial immersed in the oil bath. The crystals were kept at 185 °C overnight, and a suitable ordered crystal was selected for the SC XRD experiment. The full conversion was confirmed by matching the simulated powder XRD pattern with the experimental pattern of the ordered phase prepared from the melt.

*Preparation of the Thin Films of TPP_2_SbBr_5_ on Various Substrates*: Thin films of TPP_2_SbBr_5_ were prepared by spin‐coating from acetonitrile or dichloromethane solutions with concentration of 100 mg mL^−1^. The dried chemicals and solvents were used, but the preparation of precursor and spin‐coating were performed under ambient conditions. Furthermore, all thin film samples were stored in air and have shown no deterioration of the emissive layer from either oxygen or moisture. Film thickness was controlled by the speed of the rotation. A wide range of substrates was tested: microscope slide glass, quartz, Si, and Si/SiO_2_. The substrates were cleaned by sonication for 15 min at 50 °C subsequently in detergent (Hellmanex III)/H_2_O, deionized H_2_O, acetone, and isopropanol. After prompt drying with compressed air, substrates were treated for 15 min in plasma cleaner (Plasma Prep III solid state, RF power 100 W, pressure 150 mTorr).

### Characterization

Thickness of the films was estimated using stylus profilometer (D‐120 by KLA Tencor).

Optical images of the films were captured using a Leica DM4 M optical microscope in dark‐field, bright‐field, and fluorescence mode. In fluorescence mode, sample was excited with metal halide lamp (EL 6000, Leica) with 400 nm dichroic mirror to separate excitation and emission.

Powder XRD patterns were collected in transmission mode (Debye−Scherrer Geometry) with a STADI P diffractometer (STOE& Cie GmbH) equipped with a curved Ge (111) monochromator (Cu Kα1 = 1.54056 Å) and a silicon strip MYTHEN 1K Detector (Fa. DECTRIS). For the measurement, a ground powder was placed between adhesive tape.

Single‐crystal XRD measurements were conducted on an Oxford Xcalibur S diffractometer equipped with a Sapphire 3 CCD detector and molybdenum (Mo Kα = 0.71073 Å) sealed tube as an X‐ray source. Crystals were tip‐mounted on a micromount with paraffin oil. The data were processed with Oxford Diffraction CrysAlis Pro software; structure solution and refinement were performed with SHELXS and SHELXL, respectively, imbedded in the Olex2 package.^[^ ^81^, ^82^ ^]^ The crystal structure of the synthesized compounds was solved with direct methods.

Thermogravimetry (TG) and differential scanning calorimetry (DSC) were performed using a Netzsch simultaneous thermal analyzer (STA 449 F5 Jupiter). A powdered sample was placed in an alumina crucible with the lid and heated under Ar gas flow (50 mL min^−1^) to 230 °C (10 °C min^−1^).

Temperature dependences of PL and PLE spectra were acquired with FluoTime 300 spectrometer from PicoQuant GmbH coupled with CS204, closed cycle helium cryostat, from Advanced Research Systems. Excitation was provided from Xe lamp (power of 300W) passed through a monochromator and cut at emission monochromator with 400 nm longpass filter. The temperature of the sample was ramped with a speed of 2 K min^−1^. Spectra were corrected on the spectral sensitivity of the detector and spectral power density of the lamp.

Absolute values of PLQY were measured using a Quantaurus‐QY spectrometer from Hamamatsu in powder mode.

UV–vis absorbance spectra were obtained using the Kubelka–Munk transformation of diffuse reflectance of the microcrystalline powders that were collected using a Jasco V670 spectrophotometer equipped with a deuterium (D_2_) lamp (190–350 nm) for UV, a halogen lamp (330–2700 nm) for UV–NIR, and an integrating sphere (ILN‐725) with a working wavelength range of 220–2200 nm.

Optical absorption of thin films was measured with Jasco V770 equipped with integrating sphere ISN‐923. The sample was placed in front of integrating sphere to reduce influence of scattering. Absorption coefficient was recalculated from thickness of the film, which was measured using stylus profilometer (D‐120 by KLA Tencor).

Raman spectra were measured using a Horiba LabRAM HR Evolution confocal microscope. An objective (100× magnification, NA = 0.9) was used to induce and collect Raman scattered light from micrometer‐scale regions of the samples, using laser excitation at 785 nm (cw, 30 mW). No sample degradation was observed during or after the typical acquisition time of ≈100 s.

Scanning electron microscopy was carried out at FEI Quanta 200F FEG‐SEM (Thermo Fischer Scientific) at an acceleration voltage of 20 kV in secondary electron detection mode.

### Simulation Details

The excited state geometry calculations were carried out at the density functional theory level as implemented in the open‐source CP2K^[^ ^83^ ^]^ quantum chemistry code. A single crystallographic unit cell was used. For the disordered phase, the occupancy of one of the axial Bromine atoms (irrespective of which one as both positions are related by the inversion center) was set to 100% and the other to 0%. A mixed plane‐wave and Gaussian basis set approach was used to describe the wave function and electronic density, respectively. The kinetic energy cutoff of the plane‐wave basis was set to 400 Rydberg, while a double‐ζ basis set with polarization functions was used to describe the molecular orbitals. Scalar relativistic effects were accounted for by using effective core potential functions in the basis set. Spin–orbit coupling effects were not included. Calculations with lattice relaxation for the ground state geometry optimization were performed to account for statistical disorder in the experimental crystal structures. Unit cell parameters were taken from experimental data and not relaxed, whereas atomic coordinates were optimized until the force reached 0.023 eV Å^−1^. For the excited state geometry, the unrestricted Kohn‐Sham approach with a triplet multiplicity was used, unit cell was not optimized, while atomic coordinates were optimized until the force reached 0.023 eV Å^−1^.

The phonon density of states *g*(ω) at 300 K was analyzed via the CP2K computational package and the TRAVIS analyzer.^[^ ^84^, ^85^ ^]^ Briefly, *g*(ω) and the several partial phonon densities of states *g_i_* (ω) for selected subgroups *i* (*i* = Sb, Br, organic moiety) were obtained from the mass‐weighted Fourier transformation of an ab initio molecular dynamics (AIMD) trajectory at 300 K (within the *NPT* ensemble, a velocity rescaling thermostat with time constant of 50 fs was used) according to

(1) $$\begin{array}{c} g_{i}\;\left(\omega \right)=\;m_{i}\omega^{2}{\left|F\left\{r_{i}\left(t\right)\right\}\right|}^{2}b\left(\omega ,T\right) \end{array}$$

(2) $$\begin{array}{c} b\;\left(\omega ,T\right)=\frac{\hbar \omega }{k_{B}T}\;\left(1+{\left[e^{\hbar \omega /\left(k_{B}T\right)}-1\right]}^{-1}\right) \end{array}$$

(3) $$\begin{array}{c} g\;\left(\omega \right)=\underset{i}{\sum }g_{i}\left(\omega \right)\; \end{array}$$

where $F\{r_{i}(t)\}$ denotes a Fourier transformation of the position correlation function *r_i_* (*t*) and the term *b*(ω, *T*) converts the semiclassical mode occupation of AIMD (each mode of energy ℏω features a thermal occupation according to *k*_B_ *T*/ℏω) into a Bose–Einstein occupation. For the calculation of all phonon densities of states, care was taken to reach sufficient thermal equilibration (typically within the first 3 ps) before analyzing the AIMD trajectory.

### FLIM Thermography Imaging Experiment

As a heating element, a patterned ITO thin film on a 1 mm thick glass substrate was used. The patterning was performed by a standard photolithography process. Briefly, AZ5214E photoresist was spin‐coated on the ITO‐on‐glass substrates, baked for 120 s at 90 °C, the structure was exposed (DWL66+ by Heidelberg), baked for 120 s at 115 °C, the entire substrate was exposed (MJB3 by Karl Süss), and the structure was developed with MF319 developer for 45 s and rinsed with water. The ITO was etched with 10% hydrochloric acid solution for 30 s, rinsed with water, and the remaining resist was stripped with acetone and rinsed with 2‐propanol.

The heat distribution in the obtained ITO pattern was checked by a LWIR camera: Seek Thermal Compact Pro customized with a homemade ZnSe lensed macro‐objective made according to the project https://www.thingiverse.com/thing:525605.

Thin films in a range 150–650 nm were spin‐coated from acetonitrile 100 mg mL^−1^ precursor according to the procedure described above. The current through the ITO pattern was provided by Keithley 236 Source‐Measure Unit in the voltage stabilization mode. Picosecond laser with wavelength of 440 nm and repetition rate of 2.5 MHz was applied for the excitation of thermography images. The FLIM imaging was performed with a Leica TCS SP8 confocal microscope coupled to PicoHarp 300 TCSPC and Leica SP8 FALCON FLIM modules. Both modules after optimization of scanning speed and exposure time parameters provided similar results in the thermographic accuracy.

## Conflict of Interest

The authors declare no conflict of interest.

## Supporting information

Supporting Information
