# Supplementary material for: Hybrid 0D Antimony Halides as Air‐Stable Luminophores for High‐Spatial‐Resolution Remote Thermography
Source: Adv Mater. 2021 Jan 22;33(9):2007355. doi: 10.1002/adma.202007355 (PMC11481058; doi:10.1002/adma.202007355)
Supplement: Supplementary file 1 — Supporting Information [file ADMA-33-2007355-s001.pdf]

© 2021 Wiley-VCH GmbH

ADVANCED MATERIALS

Supporting Information

for *Adv. Mater.*, DOI: 10.1002/adma.202007355

Hybrid 0D Antimony Halides as Air-Stable Luminophores for High-Spatial-Resolution Remote Thermography

*Viktoriia Morad, Sergii Yakunin, Bogdan M. Benin, Yevhen Shynkarenko, Matthias J. Grotevent, Ivan Shorubalko, Simon C. Boehme, and Maksym V. Kovalenko**

© 2020 Wiley-VCH GmbH

Supporting Information

Highly luminescent hybrid zero-dimensional antimony halides as air-stable luminophores for high-spatial-resolution remote thermography

*Viktoriia Morad, Sergii Yakunin, Bogdan M. Benin, Yevhen Shynkarenko, Matthias J. Grotevent, Ivan Shorubalko, Simon C. Boehme, Maksym V. Kovalenko**

Materials:
Antimony(III) bromide (SbBr3, 99%, ABCR, 50g), tetraphenylpshosphonium bromide (TPPBr, 98+%, 10g, ABCR, dried under vacuum at 60 °C overnight), N,N-dimethylformamide (DMF, 99.8%, Extra Dry over Molecular Sieve, AcroSeal®), acetonitrile (>99.9% HPLC LiChrosolve gradient grade, dried using molecular sieve column packing system), dichloromethane (DCM, for HPLC, unstabilized 99.8%, dried using molecular sieve column packing system), diethyl ether (Et_2O, 99.5%, Extra Dry over Molecular Sieve, Stabilized, AcroSeal®). Dry solvents, TPPBr and $SbBr_3$ were stored in the air- free N_2-filled glovebox with H_2O and O_2 levels <0.1 ppm. The films were spin-coated in ambient atmosphere.

Table S1. Crystal data and structure refinement for disordered TPP_2SbBr_5

Empirical formula	$C_{24}H_{20}Br_{2.5}PSb_{0.5}$
Formula weight	600.02
Temperature/K	200.01(10)
Crystal system	triclinic
Space group	P-1
a/Å	10.2034(5)
b/Å	10.2266(4)
c/Å	12.4257(5)
α/°	100.053(3)
β/°	93.944(3)
γ/°	114.499(4)
Volume/Å^3	1147.22(9)
Z	2
ρ_{calc}g/cm^3	1.737
μ/mm^{-1}	5.057
F(000)	584.0
Crystal size/mm^3	0.447 × 0.38 × 0.093
Radiation	Mo Kα (λ = 0.71073)
2Θ range for data collection/°	3.37 to 57.988
Index ranges	-13 ≤ h ≤ 13, -13 ≤ k ≤ 13, -16 ≤ l ≤ 16
Reflections collected	16871
Independent reflections	5326 [R_{int} = 0.0357, R_{sigma} = 0.0406]
Data/restraints/parameters	5326/0/259
Goodness-of-fit on F^2	1.063
Final R indexes [I>=2σ (I)]	R_1 = 0.0410, wR_2 = 0.0767
Final R indexes [all data]	R_1 = 0.0607, wR_2 = 0.0847
Largest diff. peak/hole / e Å^{-3}	0.73/-1.25

Table S2. Crystal data and structure refinement for ordered TPP_2SbBr_5

Empirical formula	$C_{48}H_{40}Br_5P_2Sb$
Formula weight	1200.04
Temperature/K	297.3(8)

Crystal system	triclinic
Space group	P-1
a/Å	10.5176(6)
b/Å	10.5226(6)
c/Å	23.2420(12)
α/°	96.646(5)
β/°	100.624(6)
γ/°	108.060(6)
Volume/Å^3	2362.1(2)
Z	2
ρ_{calc}g/cm^3	1.687
μ/mm^{-1}	4.912
F(000)	1168.0
Crystal size/mm^3	0.412 × 0.228 × 0.115
Radiation	Mo Kα (λ = 0.71073)
2Θ range for data collection/°	3.628 to 50.054
Index ranges	$-12 \leq h \leq 12$, $-12 \leq k \leq 12$, $-27 \leq l \leq 27$
Reflections collected	30837
Independent reflections	8361 [R_{int} = 0.0896, R_{sigma} = 0.0994]
Data/restraints/parameters	8361/0/505
Goodness-of-fit on F^2	1.015
Final R indexes [I>=2σ (I)]	R_1 = 0.0776, wR_2 = 0.1775
Final R indexes [all data]	R_1 = 0.1602, wR_2 = 0.2240
Largest diff. peak/hole / e Å^{-3}	1.11/-0.89

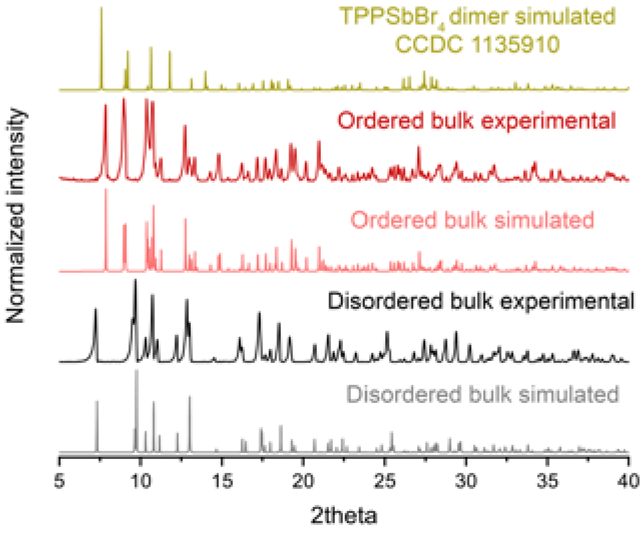

Figure S1. Comparison of the experimental and simulated XRD patterns for the disordered and ordered phases of TPP_2SbBr_5. The simulated $TPPSbBr_4$ dimer pattern (from CCDC 1135910) plotted to ensure this impurity is not present.

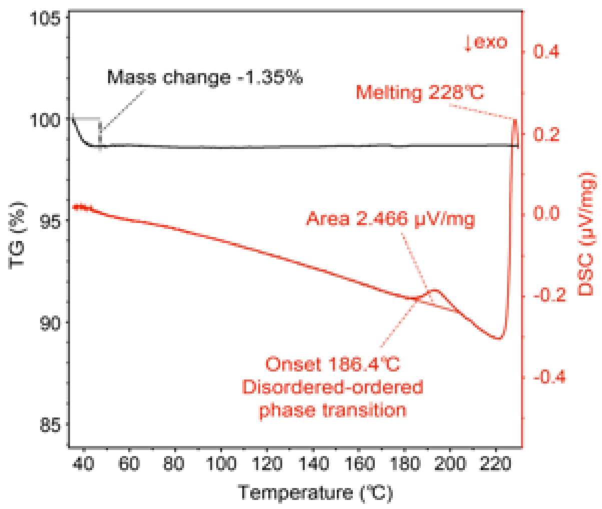

Figure S2. Thermogravimetric (TG) curve (black) and differential scanning calorimetry (DSC) curve (red) for heating of the bulk disordered TPP_2SbBr_5 powder. TG analysis reveals no mass loss throughout the experiment except 1.35% at around 40 °C which can be related to the absorbed water. DSC curve features two exothermic peaks: at 186.4 °C which indicates disordered-ordered phase transition and at about 228 °C which indicates the melting of the compound.

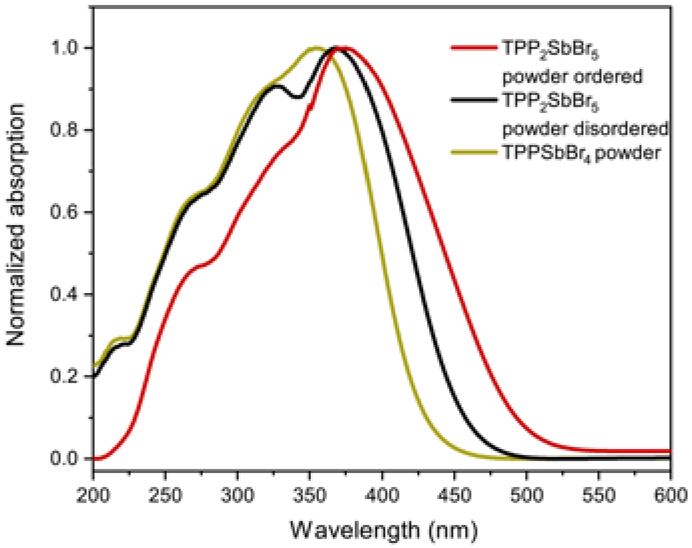

Figure S3. Kubelka-Munk transform of the diffuse reflectance spectra of TPP_2SbBr_5 ordered and disordered powdered samples as well as $TPPSbBr_4$ powdered sample.

Table S3. PLQY of TPP_2SbBr_5 in various forms, ex. wavelength 400 nm

**Compound**	**PLQY, %**
Ordered bulk	33
Ordered film	25
Disordered bulk	3.3
Disordered film	2.3

Table S4. Bond Lengths for disordered TPP_2SbBr_5

**Atom**	**Atom**	**Length (Å)**
Sb1	Br1	2.7769(4)
Sb1	Br1[1]	2.7769(4)

Table S4. Bond Lengths for disordered TPP_2SbBr_5

Atom	Atom	Length (Å)
Sb1	$Br2^1$	2.7946(5)
Sb1	Br2	2.7946(5)
Sb1	Br3	2.3765(7)
Sb1	$Br3^1$	2.3765(7)

Table S5. Bond Angles for disordered TPP_2SbBr_5

Atom	Atom	Atom	Angle (˚)
Br1	Sb1	$Br1^1$	180.0
$Br1^1$	Sb1	$Br2^1$	90.357(14)
$Br1^1$	Sb1	Br2	89.642(14)
Br1	Sb1	$Br2^1$	89.642(15)
Br1	Sb1	Br2	90.358(14)
Br2	Sb1	$Br2^1$	180.0
Br3	Sb1	$Br1^1$	95.22(2)
$Br3^1$	Sb1	Br1	95.22(2)
$Br3^1$	Sb1	$Br1^1$	84.78(2)
Br3	Sb1	Br1	84.78(2)
$Br3^1$	Sb1	$Br2^1$	89.03(2)
$Br3^1$	Sb1	Br2	90.97(2)
Br3	Sb1	Br2	89.03(2)
Br3	Sb1	$Br2^1$	90.97(2)
$Br3^1$	Sb1	Br3	180.00(3)

Table S6. Bond Lengths for ordered TPP_2SbBr_5

Atom	Atom	Length (Å)
Sb1	Br1	2.7323(16)
Sb1	Br2	2.8266(13)
Sb1	Br3	2.7905(15)
Sb1	Br4	2.7147(14)
Sb1	Br5	2.5246(13)

Table S7. Bond Angles for ordered TPP_2SbBr_5

Atom	Atom	Atom	Angle (˚)
Br1	Sb1	Br2	92.94(5)
Br1	Sb1	Br3	173.64(5)
Br3	Sb1	Br2	93.16(4)
Br4	Sb1	Br1	86.47(5)
Br4	Sb1	Br2	169.80(5)
Br4	Sb1	Br3	87.84(5)
Br5	Sb1	Br1	88.84(5)
Br5	Sb1	Br2	93.79(5)
Br5	Sb1	Br3	88.97(5)
Br5	Sb1	Br4	96.38(4)

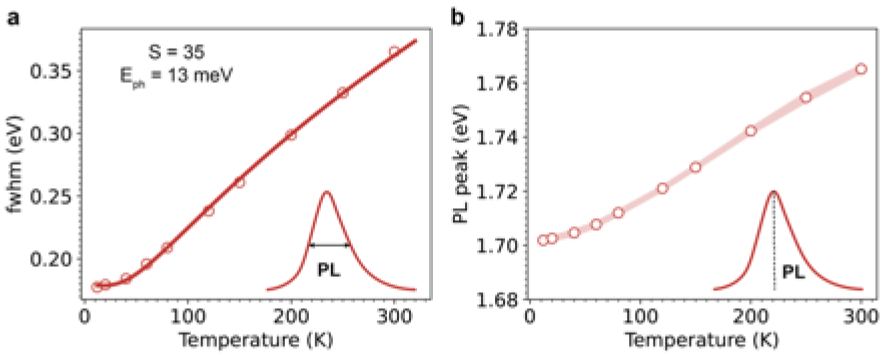

Figure S4. (a) FWHM of the PL dependence on the temperature for TPP_2SbBr_5. The FWHM values for each temperature were fitted with the Toyozawa model to obtain the S parameter and energy of phonons E_{ph}. (b) The corresponding PL peak position shift with temperature. PL peak positions and FWHMs were obtained from a skewed Gaussian model fit.

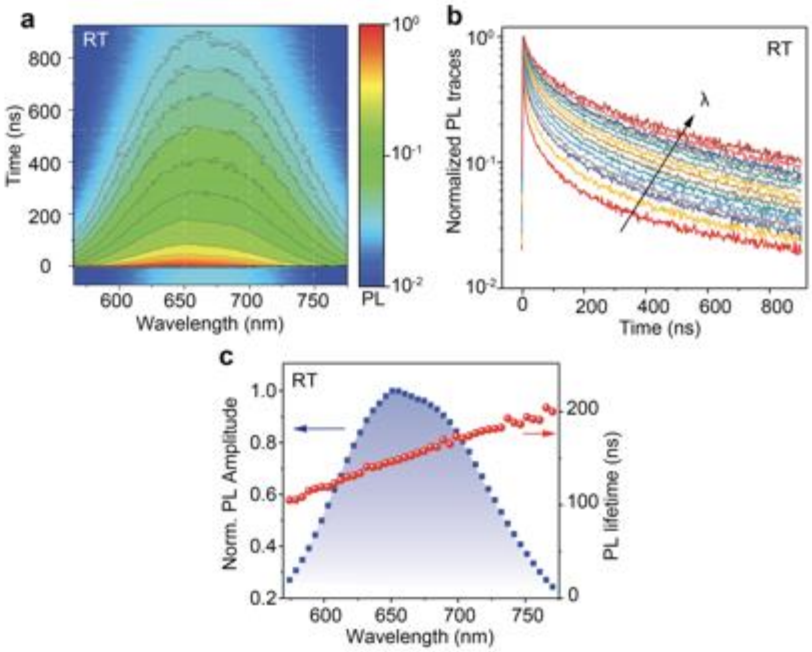

Figure S5. (a) Time-resolved emission spectra of TPP_2SbBr_5. (b) Normalized TRPL traces at different wavelength. (c) Normalized PL amplitude and PL lifetime.

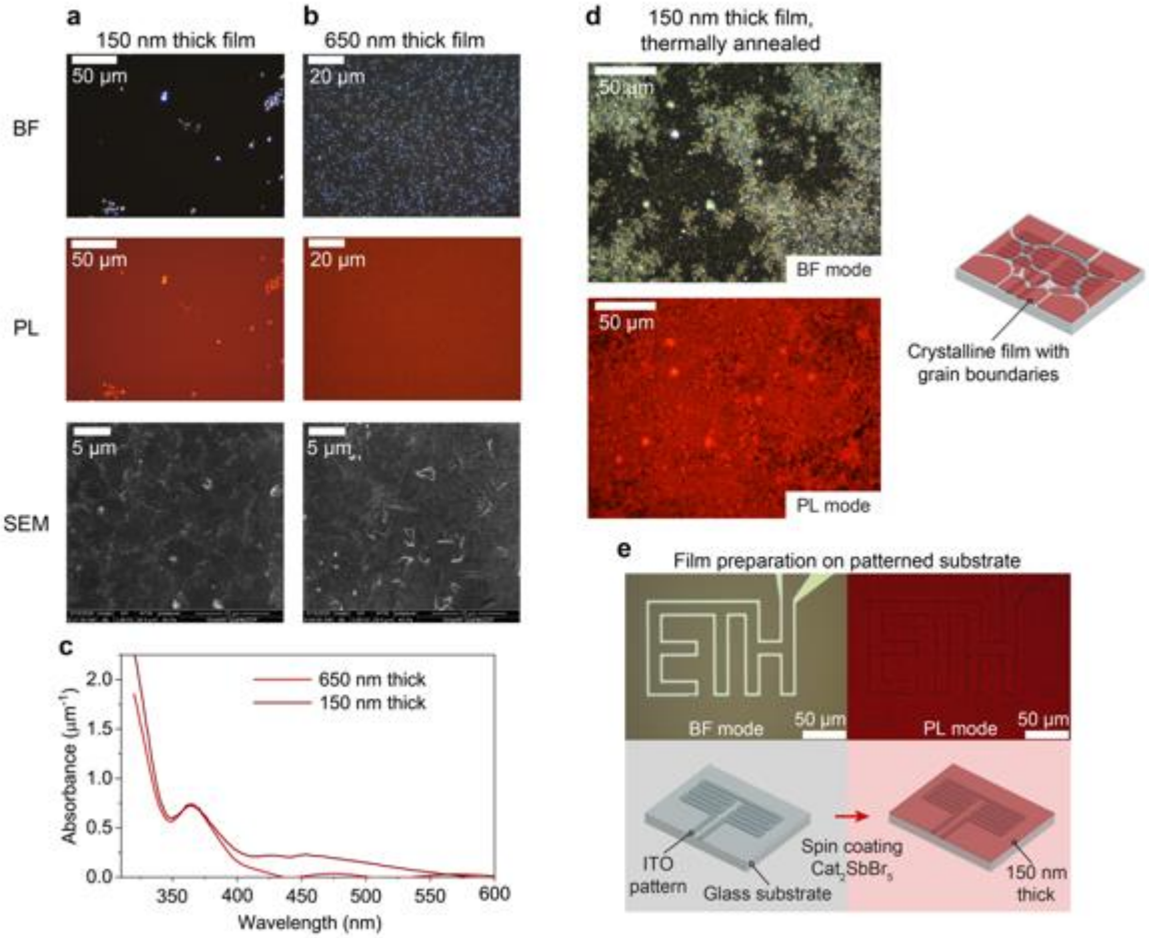

Figure S6. Optical microscope images (in bright filed and PL modes) and SEM of the (a) 150 nm and (b) 650 nm thick TPP_2SbBr_5 films prepared on glass substrates. The thinner film samples usually are more homogenous, as evidenced from optical microscope images in BF and PL modes. For thicker films, small crystallites, visible in BF as well as PL modes, are present. The SEM images with high resolution (scalebar 5 μm) for both films show similar size of crystallites, but in case of thicker film, larger grains are also present. (c) Optical absorption spectra of the resulting films on glass substrates. (d) Optical microscope images (in bright filed and PL modes) of the thermally annealed 150 nm film. (e) Glass substrate with ITO pattern (feature size 3 μm) before and after coating TPP_2SbBr_5 film of about 150 nm thickness for a confocal microscopy FLIM experiment.

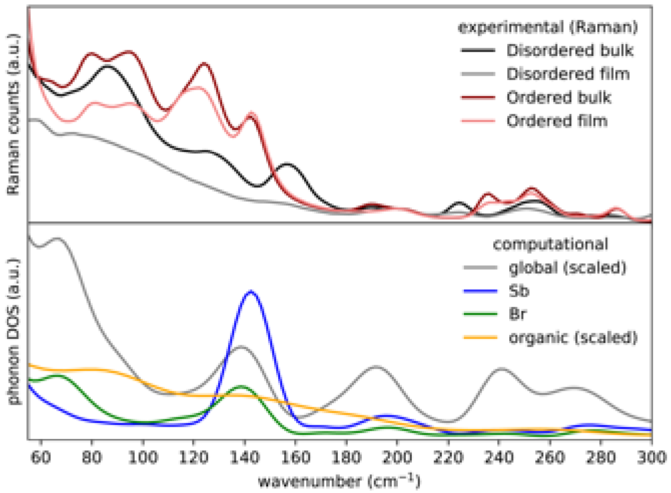

Figure S7. The comparison of the simulated (bottom) phonon density of states and experimental (top) Raman spectra of TPP_2SbBr_5 in various forms.

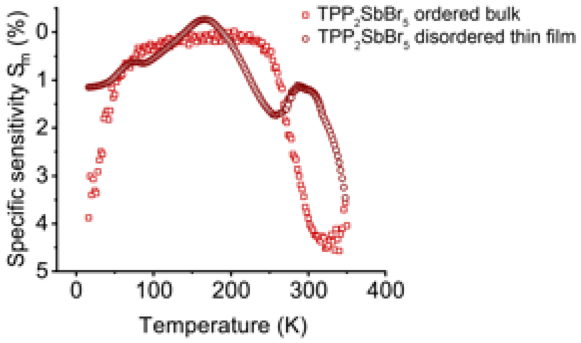

Figure S8. The specific sensitivity, $|\alpha| = -\frac{d\tau}{dT} \cdot \frac{1}{\tau}$, for the investigated thermographic phosphor, comparing the ordered bulk materials and disordered thin film.

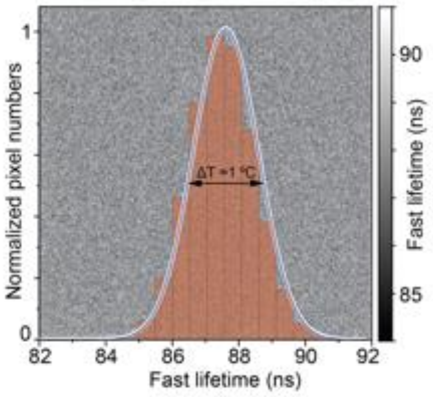

Figure S9. Fast lifetime distribution of individual pixels measured from the cold patterned substrate. The background image is a fast lifetime map, measured over the cold sample.

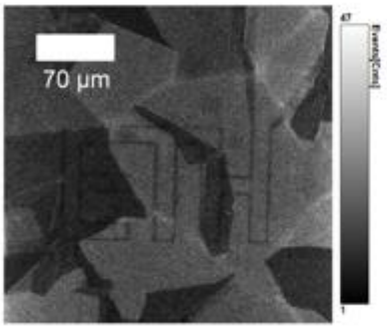

Figure S10. Confocal microscope image of the TPP_2SbBr_5 recrystallized film in PL intensity detection mode, demonstrating that grains with different degree of crystallinity have different optical properties.

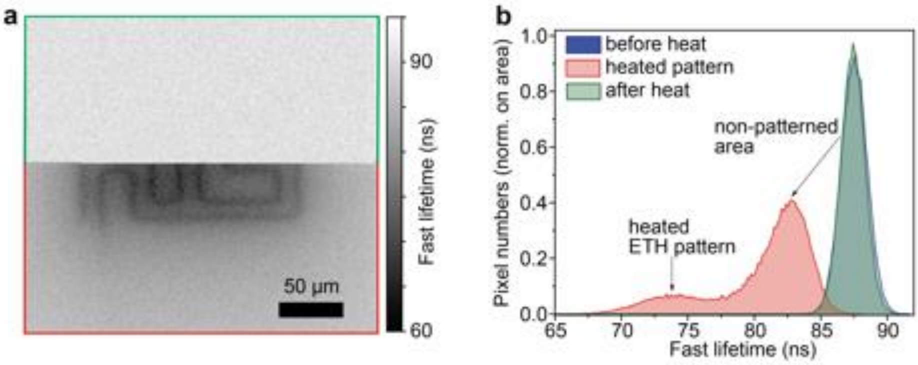

Figure S11. (a) Fast lifetime map measured over the sample with the moment of switching of the heating current, the corresponding parts of the image shown by green and red borders. (b) the corresponding fast lifetime distributions from the heated sample on and out of the ITO pattern as well as the comparison of the cold sample before and after the heating.

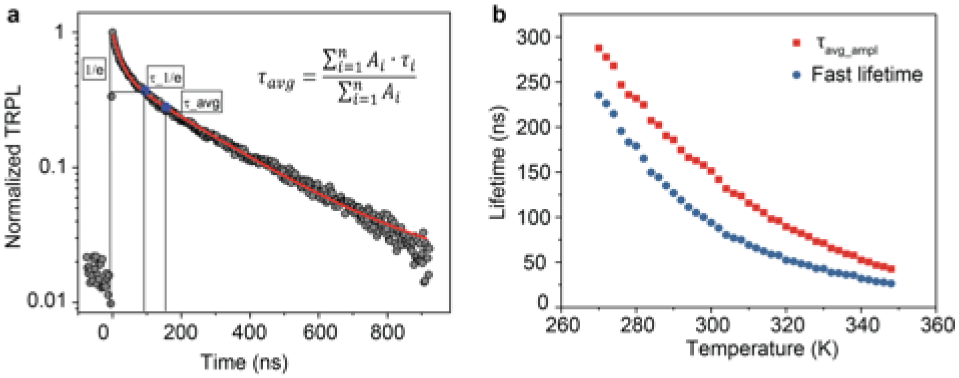

Figure S12. Illustration of lifetime estimation variation for non-monoexponential decay traces. (a) Typical TCSPC trace and the values of decay time at 1/e intensity (so-called "fast lifetime" used in FLIM setup) and value of averaged lifetime: $\tau_{avg} = \frac{\sum_{i=1}^{2} A_i \cdot \tau_i}{\sum_{i=1}^{2} A_i}$ used in non-imaging TCSPC TRPL setup; (b) the difference between temperature dependencies of lifetime values acquired as 1/e delay time (fast lifetime) and τ_{avg} fitted from the bi-exponential model.

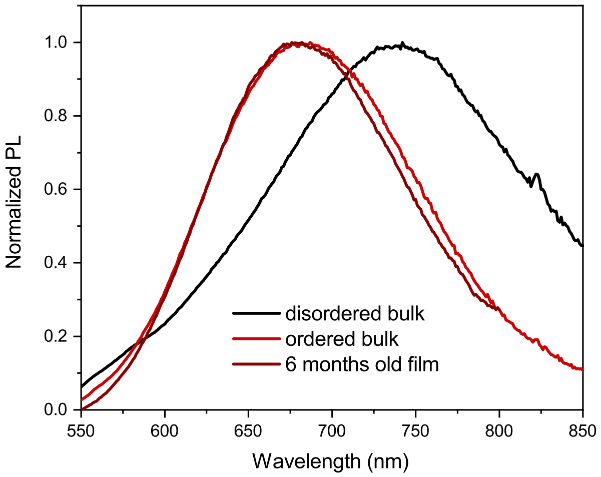

Figure S13. Illustration of the air-stability of TPP_2SbBr_5. PL spectra of the thin film (deposited on the ITO-patterned substrate as employed in Figure 4e), measured about 6 months after spin-coating. Ordered bulk and disordered bulk PL spectra are plotted for comparison. As evident, over time the film converts to a thermodynamically more stable ordered phase and remains in this phase without change of PL characteristics. These characteristics match those of the bulk sample, which confirms the air-stability of TPP_2SbBr_5.

Supplementary Note 1. Difference between the evaluation of 1/e decay time (fast lifetime) and averaged lifetime.

The non-monoexponential behavior for TRPL traces adds a certain complexity to the comparison of the PL lifetimes determined by different instruments. In the spectroscopy studies with relatively narrow data sets it is reasonable to carefully fit the individual time-resolved photoluminescence (TRPL) traces and evaluate so-called average lifetimes (amplitude-weighted) in the bi-exponential model, while in FLIM experiment data sets are sufficiently larger and thus more time-effective and also more accurate is simply to perform the evaluation of "fast lifetime" that is the time of the 1/e drop in the trace. Unfortunately, there is no fixed coefficient or analytical solution to the equation of transform one τ_{avg} and such an equation has to be solved in a numerical form.
